# Glatiramer Acetate, Dimethyl Fumarate, and Monomethyl Fumarate Upregulate the Expression of CCR10 on the Surface of Natural Killer Cells and Enhance Their Chemotaxis and Cytotoxicity

**DOI:** 10.3389/fimmu.2016.00437

**Published:** 2016-10-19

**Authors:** Azzam A. Maghazachi, Kristin L. Sand, Zaidoon Al-Jaderi

**Affiliations:** ^1^Department of Clinical Sciences, College of Medicine, and the Sharjah Institute for Medical Research (SIMR), University of Sharjah, Sharjah, United Arab Emirates; ^2^University of Oslo, Oslo, Norway

**Keywords:** NK cells, glatiramer acetate, dimethyl fumarate, monomethyl fumarate, cancer, chemotaxis, cytotoxicity

## Abstract

*In vitro* harnessing of immune cells is the most important advance in the field of cancer immunotherapy. Results shown in the current paper may be used to harness natural killer (NK) cells *in vitro*. It is observed that drugs used to treat multiple sclerosis such as glatiramer acetate, dimethyl fumarate, and monomethyl fumarate upregulate the expression of chemokines receptor 10 (CCR10) on the surface of human IL-2-activated NK cells. This is corroborated with increased chemotaxis of these cells toward the concentration gradients of the ligands for CCR10, namely CCL27 and CCL28. It is also demonstrated that these three drugs enhance NK cell cytotoxicity against tumor target cells, an activity that is abrogated by prior incubation of the cells with anti-CCR10 antibody. Because CCL27 and CCL28 are secreted by selective tumor types such as malignant melanoma, squamous cell carcinomas, and colorectal cancer, respectively, it is hypothesized that activated NK cells may be harnessed *in vitro* with any of these drugs before utilizing them as a therapeutic modality for cancer.

## Introduction

Natural killer (NK) cells perform several important functions; among them, the regulation of the adaptive immune response by secreting cytokines such as IFN-γ, shaping the innate immune system by interacting with dendritic cells, defending against viral infection, and lysing and destroying tumor cells (1–5). Resting NK cells respond to dangers occurring at sites of injury. Evidence gathered from a mouse xenograft tumor model testing functionally deficient NK cells or antibody-mediated NK cell depletion supports that NK cells can eradicate tumor cells. An 11-year follow-up study in patients indicated that low NK-like cytotoxicity was associated with increased cancer risk (6). High levels of tumor-infiltrating NK cells are associated with a favorable tumor outcome in patients with colorectal carcinoma, gastric carcinoma, and squamous cell lung cancer, suggesting that NK cell infiltration into tumor tissues represents a positive prognostic marker (7).

Chemokines are molecules that play essential roles in linking the innate and adaptive immune responses (8) and are crucial in health and diseases (9). They have low molecular weights and are divided into four subfamilies based on the position of the cysteine (C) residue in the amino terminal end of the molecules; these are known as CXC, CC, C, and CX3C. They play important roles in NK cell biology maintaining them in the bone marrow, guiding them into the circulation, and aiding their accumulation at sites of injury. The field examining the expression of chemokine receptors in NK cells, and their ability to induce their migration, started in early 1990s (10, 11). There are overwhelming results describing the effects of chemokines on various biological activities of NK cells [reviewed in Ref. (12)].

Glatiramer acetate (GA; commercial name Copaxone) is a synthetic compound made up of the four amino acids Glu, Ala, Lys, and Tyr that are most common in myelin basic protein (13). GA is a first-line immunomodulatory therapy in relapsing-remitting multiple sclerosis (RRMS) patients (14). Although the drug is not as effective as second-line therapies such as Natalizumab and Fingolimod, GA is widely used due to few serious side effects. This drug showed promise in maintenance therapy, when used after more intensive immunosuppression (15). It was also reported that GA enhanced the *in vitro* killing of autologous and allogeneic human immature and mature monocyte-derived dendritic cells (DCs) by activated human NK cells (16). Further, administration of GA into mice ameliorated the EAE clinical scores, and this was associated with high killing of dendritic cells by NK cells isolated from the same mice (17).

Dimethyl fumarate (DMF), also known as Tecfidera (Biogen, Cambridge, MA, USA), has been approved by the FDA as an oral therapy for multiple sclerosis (MS) patients due to its efficacy. The mechanism of action of DMF is not completely understood. However, it was suggested that DMF may be hydrolyzed by esterases to monomethyl fumarate (MMF), although it is not yet clear whether MMF might mediate the *in situ* effects of DMF (18). It has also been demonstrated that DMF inhibits the proliferation of A375 or M24met cell lines and reduces melanoma growth and metastasis in experimental melanoma mouse models (19).

We recently reported that MMF increased primary human CD56^+^ NK cell lysis of K562 and RAJI tumor cells (20). Moreover, MMF upregulated the expression of NKp46 on the surface of NK cells, which was correlated with upregulation of CD107a (lysosomal-associated membrane protein-1 “LAMP-1”) on the surface of CD56^+^ NK cells, and the release of Granzyme B from CD56 NK cells (20). Moreover, MMF inhibited the EAE clinical score in SJL mice correlated with enhanced NK cell lysis of dendritic cells (21).

In the present report, we describe a novel effect of GA, MMF, and DMF. We observed that these drugs upregulate the expression of CCR10 on the surface of IL-2-activated NK cells, corroborated with increased cytotoxicity, and induced chemotaxis toward the ligands for CCR10, namely CCL27 and CCL28. These observations may have implications for utilizing the highly antitumor effector NK cells in the therapy of cancer, particularly for those patients where tumor cells secrete the ligands for CCR10.

## Materials and Methods

### Reagents

FITC-conjugated mouse antihuman CCR3, CCR4, CCR5, CCR6, CCR7, CCR9, CXCR1, CXCR3, CXCR4, and CXCR5 or unconjugated monoclonal mouse-antihuman CCR1, CCR2, and CXCR6, as well as PE-conjugated rat antihuman CCR8, PE-conjugated rat antihuman CCR10, and PE-conjugated rat IgG2b, were obtained from R&D Systems Europe Ltd. (Abingdon, UK). FITC-conjugated mouse antihuman CX_3_CR1 was purchased from Medical and Biological Laboratories Co. Ltd. (Nagoya, Japan). FITC-conjugated monoclonal mouse antihuman CD3, PE-conjugated monoclonal mouse antihuman CD56, and FITC-conjugated goat anti-mouse were purchased from Becton-Dickinson (San Diego, CA, USA). FITC-conjugated mouse IgG, PE-conjugated mouse IgG, unconjugated mouse IgG, and unconjugated rat IgG were obtained from either Becton-Dickinson or from R&D Systems. Pertussis toxin (PTX), MMF, and DMF were obtained from Sigma-Aldrich (Saint Louis, MO, USA). CCL1, CCL27, CCL28, and CXCL10 were purchased from PeproTech (London, UK).

### Preparation and Culture of NK Cells

Buffy coats from normal human volunteers were obtained from the blood bank (Ulleval Hospital, Oslo). Human IL-2-activated NK cells were prepared using Histopaque-1077 (Sigma-Aldrich) and RosetteSep human NK cell enrichment cocktail (Stemcell Technologies, SARL, Grenoble, France). NK cells were negatively selected by removing cells expressing CD3, CD4, CD19, CD36, CD66b, CD123, and glycophorin A. More than 95% of these cells expressed the CD56 molecule but lacked the CD3 molecule as determined by flow cytometric analysis (Figure S1 in Supplementary Material). Purified NK cells were then placed in flasks at 1 × 10^6^/mL and 200 U/mL IL-2 (PeproTech, Rocky Hill, NJ, USA), and then incubated at 37°C in a 5% CO_2_ incubator for 5–7 days.

### NK Cell Cytotoxicity Assay

The human myeloid leukemia cell line K562 cells (CCL-243 obtained from American type culture collection “ATCC,” Manassas, VA, USA) or RAJI human lymphoma cells (CCL-214, ATCC), were used as target cells. Target cells were incubated at 1 × 10^6^ cells/mL with 5 μg/mL Calcein AM (Sigma-Aldrich) for 45 min. The cells were pelleted by centrifugation and resuspended in RPMI. To obtain total lysis, these cells were incubated in 96-well plates with 0.05% Triton X, whereas they were incubated with medium alone to obtain total viability. In other cultures, Calcein-AM-labeled cells were incubated at 37°C in a 5% CO_2_ incubator with activated NK cells at different NK target cell ratios for 4 h. The plates were centrifuged, supernatants were removed, and replaced with PBS. Fluorescence units (FUs) were measured in Cytofluor plate reader. The percentage of cytotoxicity was calculated according to the following formula: % viability = FU of targets incubated with IL-2-activated NK cells (experimental) minus FU of targets incubated with Triton X, divided by FU of targets incubated in medium only (total viability), minus FU of targets incubated with Triton X (total lysis). In the figures, the 10:1 effector:target (E:T) cell ratio is shown; however, similar results were obtained using other E:T cell ratios (2.5:1 and 5:1). Of note, the media of incubation contains RPMI plus 10% FCS. For anti-CCR10 treatment, the cells were washed and then treated with 10 μg/mL anti-CCR10 or 10 μg/mL isotype control antibody for 45 min at 4°C, washed and examined for viability. Only more than 95% viable cells were then added to Calcein-AM-labeled target cells and incubated for 4 h in the NK cytotoxicity assay.

### *In Vitro* Chemotaxis Assay

Nucleopore blind well chemotaxis chambers with a lower well volume of 200 μL were used. A maximum volume of 200 μL medium containing RPMI plus 2% FCS was placed in the lower wells in the presence or absence of various chemokines. Cells (2 × 10^5^) were placed in the upper compartments and incubated for 2 h at 37°C in a 5% CO_2_ incubator separated by polycarbonate filters (Nucleopore Polycarbonate 13 mm size 8 UM, Whatman International Ltd., Kent, UK). Filters were removed, dehydrated, stained with 15% modified Giemsa stain for 7 min, and then mounted on glass slides. Cells in 10 high power fields were counted and averaged for each sample. Migration index (MI) was calculated as the number of cells migrating toward the concentration gradients of chemokines divided by the number of cells migrating toward medium only as previously described (22). For pretreatment with PTX, NK cells (1 × 10^6^/mL) were either left intact or were treated for 2 h at 37°C with 100 ng/mL-activated PTX, as previously described (22). Only more than 95% viable cells were examined.

### Flow Cytometric Analysis

IL-2-activated NK cells were either left intact or incubated with various concentrations of GA, MMF, or DMF for 24 h. The cells were washed and incubated in a 96-well plate (v-bottom, 2 × 10^5^ cells per well), washed again, and resuspended in PBS buffer containing 0.1% sodium azide and 2% fetal bovine serum. Cells were first checked for viability with Trypan blue exclusion test, and only more than 95% viable cells were used in the assay. These viable cells were labeled with antibodies for 45 min in the dark at 4°C, washed twice, and examined in the flow cytometer (FACSC II, Becton-Dickinson Biosciences, San Jose, CA, USA). Markers were set according to the isotype control FITC- or PE-conjugated mouse IgG. More than 95% viable NK cells were also incubated at 4°C in the dark with FITC-conjugated anti-CD107a or FITC-conjugated IgG1 isotype control (Becton-Dickinson Pharmingen, San Diego, CA, USA). They were washed twice and examined in the flow cytometry. Gating was performed according to the isotype control. Analysis was done by FlowJo (Flow cytometry analysis software, Ashland, OR, USA).

### Cell Lysis and Immunoblotting

More than 95% viable NK cells treated with 100 ng, 1 μg, or 10 μg GA for 24 h were lysed with ice-cold Non-idet P-40 lysis buffer containing 1% Non-idet P-40, 30 mM Tris–HCl, pH 7.4, 150 mM NaCl, 10 mM NaF, 1 mM EDTA, 10 mM sodium pyrophosphate, 1 mM sodium orthovanadate, 1 mM phenylmethyl-sulfonyl fluoride (PMSF), and a protease inhibitor cocktail (Sigma-Aldrich). The solutions were centrifuged at 13,000 × *g* for 15 min at 4°C to separate the cell lysates from the supernatants. Total protein concentration in the samples was determined using Bio-Rad Protein Assay (Bio-Rad, Uppsala, Sweden), and samples with 20-μg protein were used for each well in the gel. The samples were run on SDS-PAGE criterion gels and then electro transferred to PVDF-membranes (Millipore, Bedford, MA, USA). The membranes were blocked with 5% skim milk in TBS for 1 h, and then incubated overnight at 4°C with 1:250 dilution of polyclonal rabbit antihuman CCR10 or 1:8000 dilution of polyclonal rabbit anti-GADPH (Both from Abcam, Cambridge, UK). The membranes were washed four times in TBS-T-buffer, before incubation with HRP-conjugated secondary antibody (1:2500) (Bio-Rad) for 1 h at room temperature. Binding of antibodies to the target proteins was detected by secondary HRP-labeled antibodies and Super Signal West Pico Stable Peroxide Solution (Pierce, Rockford, IL, USA), using chemiluminescence film and CURIX 60 (Agfa HealthCare, Mortsel, Belgium).

### Detection of CCL27 (CTACK) and CCL28 (MEC) by ELISA

Natural killer cells (1 × 10^6^/mL) were incubated with 100 ng, 1 μg, or 10 μg of GA, or with culture medium as a control for 24 h at 37°C in 5% CO_2_ incubator. After incubation, the cells were harvested, and the cell suspensions were centrifuged at 1000 *g* for 8 min. Supernatants were collected and stored at −80°C until further analysis. Levels of chemokine were measured using ELISA kits from RayBiotech Inc. (Norcross, GA, USA). Color intensity was measured at 450 nm in a BioTek PowerWave XS plate reader. The standard curves and concentrations were calculated using Gen5 Data Analysis Software (BioTek Instruments, Winooski, VT, USA).

### Detection of Granzyme B by Flow Cytometric Analysis

IL-2-activated NK cells (1 × 10^6^/mL) were left intact for 24 h. Supernatants were collected from these cells and were kept at −80°C until use. To detect the expression of Granzyme B (GrB), the cells (1 × 10^6^/mL) were incubated with either media alone or with 10 μg/mL of GA overnight. In addition, the cells treated as above (with or without GA) were preincubated with supernatants collected from activated NK cells either alone or with 1 μg/mL mouse antihuman CCL27 or mouse antihuman CCL28 (R&D systems), and as a control mouse IgG1.

To stain the intracellular GrB, cells were incubated with 10 μg/mL Brefeldin A (Sigma-Aldrich) for 4 h. They were then fixed with 4% paraformaldehyde for 15 min at 4°C and then washed twice with SAP buffer (PBS with 0.1% Saponin and 0.05% NaN3) before staining intracellularly with either PE-conjugated mouse antihuman Granzyme B or isotype control PE-conjugated mouse IgG antibody (both from ImmunoTools, Friesoythe, Germany) in the dark at 4°C for 45 min. Cells were washed with flow cytometric medium and resuspended with PBS in 5-mL tubes to perform flow cytometric analysis. Gating was done according to the PE-conjugated isotype control antibody. Analysis was done by FlowJo (flow cytometry analysis software, Ashland, OR, USA).

### Statistical Analysis

Significant values were generated using several tests. We used one-way ANOVA, two-way ANOVA with Bonferroni *post hoc* correction, or the Student’s *t*-test. A *P* value <0.05 was considered to be statistically significant.

## Results

### GA Upregulates the Expression of CCR10 on the Surface of IL-2-Activated NK Cells and Induces Their Chemotaxis Toward Its Ligands

Natural killer cells migrate into inflammatory sites aided by sets of chemokine receptors, which direct them toward the chemokines present at tumor growth sites. To this end, we investigated the expression of CCR1, CCR2, CCR3, CCR4, CCR5, CCR6, CCR7, CCR8, CCR9, CCR10, CXCR1, CXCR2, CXCR3, CXCR4, CXCR5, CXCR6, CXCR7, and CX_3_CR1 on the surface of IL-2-activated NK cells after 24 h incubation with 1 or 10 μg/mL of GA. Only more than 90% viable cells as determined by Trypan blue exclusion test were used in this and subsequent assays. The reasons for choosing activated NK cells are due to the fact that at, inflammatory sites such as the tumor microenvironment, inflammatory molecules including cytokines and chemokines are released, which activate NK cells. In addition, activated NK cells migrate toward sites of inflammation much more efficiently than non-activated cells [reviewed in Ref. (12)].

**Figure 1:**
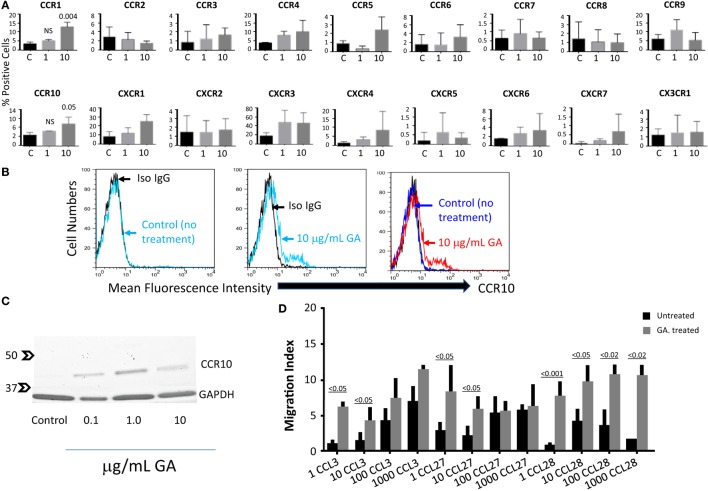
**GA upregulates the expression of CCR10 on the surface of NK cells**. **(A)** NK cells (1 × 10^6^/mL) were preincubated overnight with either media (C = control) or with 1 or 10 μg/mL of GA. The cells were washed and the expression of CCR1, CCR2, CCR3, CCR4, CCR5, CCR6, CCR7, CCR8, CCR9, CCR10, CXCR2, CXCR2, CXCR3, CXCR4, CXCR5, CXCR6, CXCR7, and CX3CR1 was determined by flow cytometric analysis. Mean ± SEM of three experiments. *P* values were determined using Student’s *t*-test. **(B)** Representative experiment showing the expression of CCR10 on the surface of IL-2-activated NK cells after incubating with media alone (control) or 10 μg/mL of GA. Gating was done according to the PE-conjugated isotype control antibody. The right panel shows an overlay combined results of the first two panels. **(C)** Immunoblot analysis showing the expression of CCR10 after 24-h pretreatment with media (control) or with 0.1, 1, or 10 μg/mL of GA. The expression of housekeeping GAPDH protein is also shown. Numbers to the left represent the molecular weights. **(D)** NK cells migrate toward the concentration gradients of CCR10 or CCR1 ligands. Various concentrations ranging between 1 and 1000 ng/mL of CCL3, CCL27, or CCL28 were placed in the lower wells of Boyden chambers, whereas 1 × 10^5^ NK cells either untreated (black columns), or pretreated for 24 h with 10 μg/mL of GA (gray columns), were placed in the upper wells. Two hours later, the filters were collected, the cells fixed, and then stained with modified Giemsa stain. Migration index was calculated as the number of cells migrating in the presence of the chemokine divided by the number of cells migrating in its absence (C = control). A comparison of migration toward CCL3, CCL27, and CCL28 among untreated NK cells (black columns) and GA-treated cells (gray columns) was performed using two-way ANOVA test of six experiments.

From all the chemokine receptors examined, GA upregulated the expression of CCR1 and CCR10 on the surface of activated NK cells (Figure 1A). Also shown are histograms where CCR10 is expressed on activated NK cells after incubating overnight with 10 μg/mL of GA (Figure 1B). We focused the rest of this work on the expression of CCR10, since another drug used for treating MS patients, namely DMF also upregulates similar expression (also see Figure 4). To confirm the upregulation of CCR10 after GA stimulation, we performed immunoblot analysis. Results in Figure 1C demonstrate that overnight incubation with 0.1, 1, or 10 μg/mL of GA induced the expression of this receptor on activated NK cells, despite the fact that flow cytometric analysis did not show increase in expression of this receptor after incubation with 1 μg/mL of GA (Figure 1A).

Consequently, we examined the chemotaxis of activated NK cells treated for 24 h with 10 μg/mL GA toward various concentrations (ranging from 1 to 1000 ng/mL) of CCL27 and CCL28, the ligands for CCR10. Cells untreated with GA migrated with low intensity toward high concentrations of CCL27 (100 and 1000 ng/mL) and CCL28 (10 and 100 ng/mL), which could be due to the low number of CCR10 positive NK cells. However, cells treated with GA for 24 h migrated toward much lower concentrations of CCL27 and CCL28 (Figure 1D). When compared to untreated cells, the migration of cells toward the lower concentrations of these chemokines was enhanced by twofold to fivefold after treatment with GA, an enhancement that was statistically significant (Figure 1D). As a control, we also observed that these cells migrated toward the concentration gradients of CCL3, one of the ligands for CCR1 after treatment with GA (Figure 1D), which is corroborated with increased CCR1 expression on the surface of these cells after similar treatment.

### Pertussis Toxin Inhibits the Chemotaxis of NK Cells

**Figure 2:**
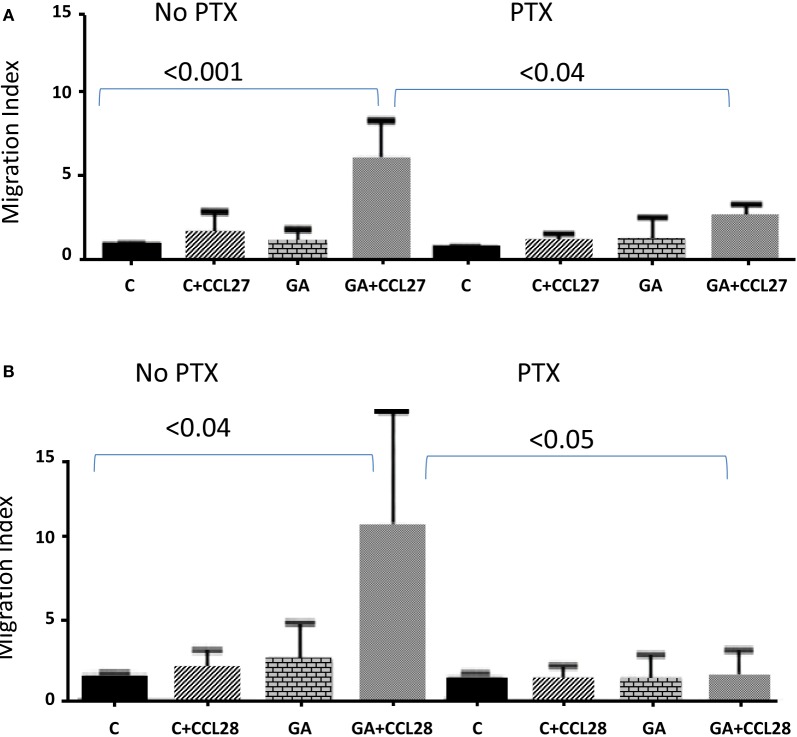
**Pretreatment with pertussis toxin (PTX) inhibits GA activity**. **(A)** Migration of cells either left untreated (C = control) or pretreated overnight with 10 μg/mL of GA toward 10 ng/mL CCL27. Before the assay, these cells were either left intact (left columns) or intoxicated with 100 ng/mL of PTX for 2 h (right columns). **(B)** Migration of cells either untreated (C = control) or pretreated overnight with 10 μg/mL of GA toward 1 ng/mL CCL28. Before the assay, these cells were either left intact (left columns) or intoxicated with 100 ng/mL of PTX for 2 h (right columns). *P* values comparing the migration of cells toward chemokines, and its inhibition by pretreatment with PTX, are determined by two-way ANOVA test of six different experiments. C = control. C+ chemokine = untreated cells migrating toward the chemokine. GA = cells treated with GA overnight and migrated toward media only (no chemokine). GA+ chemokine = cells treated with GA overnight and then migrated toward the chemokine.

We also investigated whether G proteins might be involved in mediating the chemotactic response. Consequently, activated NK cells were either left intact or were pretreated with PTX for 2 h, which intoxicates and inhibits the function of G_i_/G_o_ (22). As shown in Figure 2A, NK cells migrated toward 10 ng/mL CCL27 after preincubating for 24 h with 10 μg/mL GA (*P* < 0.001, as compared to the control). This effect was inhibited after preincubating NK cells with PTX (*P* < 0.04). Similarly, 1 ng/mL of CCL28 induced the chemotaxis of NK cells pretreated overnight with GA (*P* < 0.04, as compared to the control), and this activity was abrogated upon pretreating the cells with PTX (*P* < 0.05, Figure 2B).

### GA Enhances NK Cell Lysis of Tumor Cells: Inhibition by Anti-CCR10 Antibody

**Figure 3:**
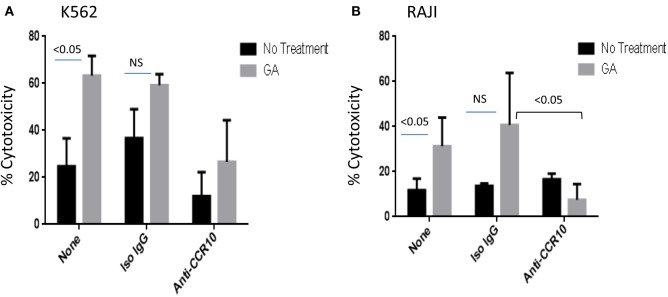
**Anti-CCR10 inhibits NK cell lysis of tumor target cells**. **(A)** NK cells were either left untreated (black columns) or incubated with 10 μg/mL of GA overnight (gray columns). Before the assay the cells were incubated for 45 min with isotype control IgG antibody or with 10 μg/mL of anti-CCR10, washed, and then mixed with Calcein-AM-labeled K562 cells in the 4-h NK cytotoxicity assay. **(B)** This is similar to **(A)** except that RAJI tumor cells were used as targets instead of K562 cells. Mean ± SEM of four separate experiments.

To evaluate whether GA might induce activities other than chemotaxis in NK cells, we performed the cytotoxicity assay. Results shown in Figure 3 demonstrate that incubating NK cells overnight with 10 μg/mL of this drug increased activated NK cell lysis of K562 cells (Figure 3A), or RAJI cells (Figure 3B). Because GA upregulates the expression of CCR10 on the surface of NK cells, we sought to demonstrate whether such expression might influence the cytolytic activity of these cells. NK cells either untreated or incubated with 10 μg/mL anti-CCR10, or as a control with isotype control IgG antibody, were examined for their ability to lyse tumor target cells in the NK cytotoxicity assay. We observed that anti-CCR10, and not the isotype control IgG, tended to inhibit NK cell lysis induced by GA of K562 (Figure 3A), but significantly inhibited lysis of RAJI cells (*P* < 0.05, comparing killing in the presence of anti-CCR10 to isotype control IgG, Figure 3B). Of note, cells treated overnight with GA and then incubated with isotype control IgG antibody also lysed K562 cells (Figure 2A) or RAJI cells (Figure 2B), but this did not reach statistical significance when compared to untreated cells incubated with the isotype control antibody.

### IL-2-Activated NK Cells Secrete CCL27 and CCL28

Because anti-CCR10 inhibited GA-induced NK cell cytotoxicity, we entertained various possibilities to explain such activity. First, we sought to determine whether GA might induce the release of CCL27 or CCL28 from activated NK cells, and that these chemokines might facilitate NK cell cytolytic activity by binding CCR10. Our results demonstrate that IL-2-activated NK cells released an average of 280 pg/mL of CCL27 (Figure S2A in Supplementary Material), or 500 pg/mL CCL28 (Figure S2B in Supplementary Material). However, addition of various concentrations ranging from 0.1 to 10 μg/mL of GA to IL-2-activated NK cells did not significantly affect the levels of these chemokines, although a trend in increased release of CCL28 was noted after pretreatment with 10 μg/mL GA (Figure S2 in Supplementary Material).

Next, we examined whether GA or a combination of GA with CCL27 or CCL28 might induce the expression of CD107a on the surface of activated NK cells. Histograms of flow cytometric analysis showed a trend of increased CD107a expression after incubating the cells with GA, CCL27, or CCL28 (Figure S2C in Supplementary Material, left). Combining GA with CCL27 or CCL28 did not augment such expression when compared to cells incubated with GA alone (Figure S2C in Supplementary Material, right). When three or more experiments were performed, the trend of increased expression of CD107a was also noticed after incubating the cells with GA, CCL27, or CCL28. However, such enhancement did not reach significant levels after treating the cells with GA, any of the two chemokines, or GA plus the chemokines (Figure S2D in Supplementary Material).

### GA or Supernatants Collected from IL-2-Activated NK Cells Induce the Expression of Granzyme B in NK Cells: Inhibition by Anti-CCL28

To further investigate the plausible mechanisms of CCR10/chemokines axis, we measured the expression of Granzyme B in activated NK cells. For this, NK cells were either incubated with media alone or with 10 μg/mL of GA overnight, and the expression of Granzyme (GrB) was detected by flow cytometric analysis. Such treatment increased the percentages of cells expressing GrB from 6 to about 30% after pretreatment with the drug (Figure S3 in Supplementary Material). Supernatants collected from activated NK cells, when added to NK cells, also increased such expression to about 12%. The enhancement of GrB expression by the supernatants was lowered by pretreatment with anti-CCL28 to about 6%, but not with anti-CCL27, whereas the isotype control for the anti-CCL27 and anti-CCL28 also did not affect the activity of the supernatants (Figure S3 in Supplementary Material, upper panels). Addition of the supernatants to GA did not result in any synergy among their activities. However, anti-CCL28, and to a lesser extent anti-CCL27, reduced the effect of GA on GrB expression (Figure S3 in Supplementary Material, lower panels).

### DMF or MMF Also Upregulates the Expression of CCR10 on the Surface of IL-2-Activated NK Cells and Induces Their Chemotaxis

**Figure 4:**
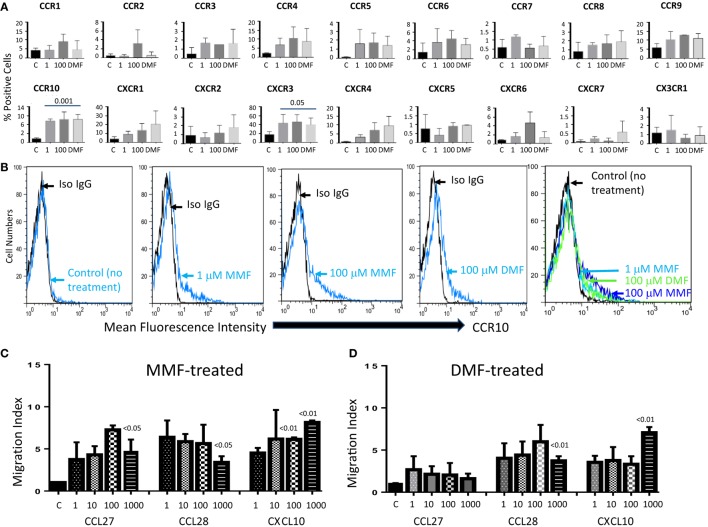
**MMF or DMF upregulates the expression of CCR10 and CXCR3 on NK cells and induces their chemotaxis toward the corresponding chemokines**. **(A)** NK cells (1 × 10^6^/mL) were preincubated overnight with either media (C = control) or with two different concentrations of MMF (1 and 100 μM) or with 100 μM DMF. The cells were washed, and the expression of CCR1, CCR2, CCR3, CCR4, CCR5, CCR6, CCR7, CCR8, CCR9, CCR10, CXCR2, CXCR2, CXCR3, CXCR4, CXCR5, CXCR6, CXCR7, and CX3CR1 was determined by flow cytometric analysis. *P* values were determined using Student’s *t*-test of three experiments comparing the percentages of CCR10^+^ or CXCR3^+^ NK cells after treatment with MMF or DMF to the controls. **(B)** Representative experiment showing the expression of CCR10 on the surface of IL-2-activated NK cells after incubating with media alone (control), 1 or 100 μM MMF, or 100 μM DMF. Gating was done according to the PE-conjugated isotype control antibody. The right panel shows an overlay combined results of the first four panels. **(C)** NK cells migrate toward the concentration gradients of CCR10 or CXCR3 ligands. Various concentrations ranging between 1 and 1000 ng/mL of CCL27, CCL28, or CXCL10 were placed in the lower wells of Boyden chambers, whereas 1 × 10^5^ NK cells either untreated (C = control) or pretreated with 100 μM MMF were placed in the upper chambers. Two hours later, the filters were collected, the cells fixed, and then stained with modified Giemsa stain. Migration index was calculated as the number of cells migrating in the presence of the chemokine divided by the number of cells migrating in its absence (C = control). A comparison of migration toward CCL27, CCL28, or CCL3 among untreated NK cells (black columns) and MMF-treated cells was performed using two-way ANOVA test of six experiments. **(D)** This is similar to **(C)**, except that 100 μM DMF was used instead of MMF.

To determine whether other drugs or metabolites used to treat or have potential for treating MS or cancer patients might affect the expression of CCR10 on NK cells similar to GA, we investigated the effects of various drugs. We observed that none of the concentrations of vitamin D_3_, its metabolite Calcipotriol, or FTY720 upregulated the expression of CCR10 on the surface of activated NK cells (not shown). In contrast, two different concentrations of MMF (1 and 100 μM) or the 100 μM concentration of DMF significantly upregulated the expression of CCR10 on the surfaces of NK cells (Figure 4A). MMF and the drug DMF also upregulated the expression of CXCR3, but not any other chemokine receptor examined (Figure 4A). Histograms of one representative experiment showed that 1 and 100 μM of MMF or 100 μM of DMF upregulated the expression of CCR10 on the surface of human IL-2-activated NK cells when compared to cells unstimulated with the drugs (Figure 4B). To corroborate the expression of CCR10 with functional activity, we performed the chemotaxis assay. Treatment of NK cells with MMF for 24 h enhanced their chemotaxis toward CCL27, CCL28, and CXCL10, the ligand for CXCR3 (Figure 4C). These results correlated well with increased expression of CCR10 and CXCR3 on NK cells after incubation with 1 or 100 μM MMF. Surprisingly, pretreatment of IL-2-activated NK cells with DMF did not increase their chemotaxis toward CCL27. However, their chemotaxis was increased toward 1 μg/mL of CCL28 or CXCL10 after overnight incubation with 100 μM DMF (Figure 4D).

### Anti-CCR10 Inhibits MMF or DMF-Induced NK Cell Lysis of Tumor Target Cells

**Figure 5:**
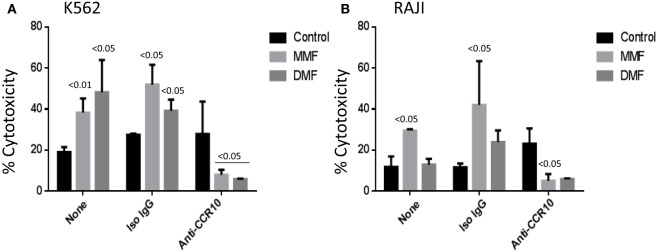
**MMF or DMF augments activated NK cell cytotoxicity against tumor cells: inhibition by anti-CCR10 antibody**. **(A)** NK cells were either left intact (black columns) or incubated with 100 μM MMF or DMF overnight (gray columns), washed, and then incubated with K562 cells. **(B)** is similar to **(A)** except that RAJI cells were used in the NK cytotoxicity assay. For treatment with anti-CCR10, the cells were washed and incubated with either 10 μg/mL of anti-CCR10 or isotype control IgG antibody for 45 min before incubating with the target cells. Percent cytotoxicity was then measured, and significant values as compared to the control in each treatment are shown on top of the columns. Significance of cytotoxicity in the presence of anti-CCR10 was compared to the cytotoxicity in the presence of isotype control IgG antibody. Mean ± SEM of four experiments.

We recently reported that MMF enhanced freshly isolated NK cell lysis of tumor cells (20). Because of the differences in the expression of NK cytotoxicity receptors among naive vs. activated NK cells, we sought to investigate whether MMF or DMF might enhance activated NK cell lysis of tumor cells. Pretreatment with either MMF or DMF enhanced IL-2-activated NK cell killing of K562 tumor cells (Figure 5A). However, only pretreatment with MMF, and not DMF, augmented activated NK cell lysis of the B cell lymphoma RAJI cells (Figure 5B). These results mirrored our recent findings showing that MMF, and not DMF, increased lysis of naive NK cells against RAJI cells (20).

Next, we asked whether CCR10 might play a role in MMF or DMF augmentation of NK cell cytotoxicity. Similar to its effect on GA-enhanced NK cell cytotoxicity, incubating activated NK cells with anti-CCR10 for 45 min abrogated the cytotoxicity induced by MMF or DMF against K562 cells (Figure 5A) or MMF-enhanced activated NK cell cytotoxicity against RAJI tumor cells (Figure 5B). This effect is not related to a general toxic effect of anti-CCR10 antibody as more than 95% of the cells were viable after treatment for 45 min with the antibodies.

## Discussion

Natural killer cells stand at the cross road among treatment of autoimmune diseases and immunodeficient diseases. In autoimmune diseases, the best protocol is to suppress the immune system, which is achieved by immunosuppressive drugs. On the other hand, in immunodeficient diseases, such as cancer or AIDS, the immune system must be activated to fight cancer cells or virally infected cells, respectively. From a first look, it appears that these methods of treatment are contradictory to each other, i.e., immunosuppression vs. immunostimulation. However, NK cells can be used as a therapeutic modality for both autoimmune diseases and cancer (23). Although there are more than 600 NCI approved clinical trials using NK cells (http://www.cancer.gov/search/results) to treat various forms of cancer, investigators are faced with the problems of targeting these highly antitumor effectors toward the sites of tumor growth.

Given the fact that NK cells can kill tumors, several strategies for the therapeutic use of NK cells have been proposed and tried in a clinical context. Cytokines have been used in the treatment of some human cancers and, in some instances, the mechanisms of action are through direct or indirect activation of NK cells (24). NK cell differentiation and activation is affected by cytokines such as IL-2, IL-12, IL-15, IL-18, IL-21, and IFNs. Several clinical trials have assessed the effects of IL-2 administration on activation and expansion of NK cells in patients with cancer (25). Apart from specific cytokines and/or growth factors, broad activators of immune function that are used in cancer treatment may also act on NK cells (25). Similarly, NK cells may have a role in the clinical efficacy of *Mycobacterium bovis* bacillus Calmette–Guérin (BCG) treatment of bladder cancer (26), indicating that mediators, which activate endogenous NK cells, can induce immune-mediated control of cancer. The potential efficacy of NK cells in treating various forms of cancer has been extensively reviewed (27–33).

CCL27 (CTACK) is expressed in epidermal keratinocytes and is responsible for attracting CCR10^+^ T cells into the skin (34, 35). On the other hand, CCL28 is expressed by epithelial cells of mucosal sites (36–38) and is upregulated in inflamed tissues such as colon, duodenal mucosa, lungs, and liver ducts (39–41). Further, treatment of Crohn’s disease patients resulted in reduced expression of CCR10 corroborated with reduced inflammation in those patients [reviewed in Ref. (42)].

Intratumoral administration of CCR10 as well as other chemokine-expressing adenoviral vector into tumor-bearing animals resulted in the recruitment and activation of T cells at sites of tumor growth (43). Similarly, transfecting CCL27 into ovarian carcinoma cells resulted in reduced tumor growth by antitumor immune responses (44). Human keratinocyte-derived skin tumors can evade T cells antitumor activity by downregulating the expression of CCR10. Further, *in vivo* work demonstrated that neutralization of CCL27 decreased leukocyte recruitment toward cutaneous tumor sites, resulting in enhanced tumor growth (45).

In contrast, it was observed that overexpression of CCR10 and CCR7 resulted in severe outcome of human cutaneous melanoma growth, as determined by high risk of relapse and death of patients (46). Along these lines, Kai et al. (47) observed that CCR10 and CCL27 were strongly expressed in human cutaneous squamous cell carcinoma, which is advantageous for tumor cell survival and proliferation. Further, hypoxic tumor cells released CCL28 recruiting CCR10^+^ Treg cells, which promote tumor growth and angiogenesis, an activity that was abrogated by anti-CCL28 (48).

To this end, we observed that GA upregulates the expression of CCR10 on the surface of human NK cells. This activity corroborated with enhanced migration of these cells toward the concentration gradients of CCL27 and CCL28, the ligands for CCR10. PTX inhibited the chemotactic effects induced by chemokines, suggesting that G protein-coupled receptors (GPCRs), and in particular G_i/o_, are involved. PTX also inhibited the cytolytic activity of NK cells induced by GA against tumor cells. Because chemokines bind GPCRs, we entertained the possibility that CCR10 could be involved in mediating NK cell cytotoxicity. We observed that anti-CCR10 reduced NK cell lysis of tumor target cells. Although the reasons behind such enigmatic effect are not clear, it is plausible that a complex interaction takes place resulting in activating the lytic potential of NK cells. It is conceivable that CCR10 upregulated by GA might interact with the CCR10 ligands such as CCL27 and CCL28 secreted by activated NK cells. Such binding may result in inducing intracellular signaling molecules that may mediate the cytolytic activity of NK cells. Hence, anti-CCR10 or PTX might interfere with such interaction resulting in inhibiting NK cells mediating lysis of tumor cells. Our results did not detect any increases in the levels of CCL27 or CCL28 secreted by NK cells after stimulation with GA. Neither there was a synergy among GA and these chemokines in the upregulation of CD107a on the surface of these cells. This molecule is considered an activation marker for NK cell-mediated lysis of tumor cells, and its expression is increased after incubating resting NK cells with MMF (20). Further analysis showed that supernatants collected from activated NK cells increased the percentages of Granzyme B positive cells, and that such activity was inhibited by pretreatment with anti-CCL28 indicating that CCL28 secreted by activated NK cells might bind CCR10 and consequently, upregulate the expression of lytic molecules on higher numbers of NK cells. GA also increased the percentages of Granzyme B positive NK cells, and this effect was reduced by anti-CCL28. Albeit not examined, it is plausible that GA might also utilize CCL28, perhaps to increase the expression of GrB. Although we have not seen a significant increase of CCL28 release after pretreatment with GA, we noted a trend of such enhancement.

Similar to GA, MMF and DMF also upregulated the expression of CCR10 on the surface of activated NK cells. Further, these chemicals enhanced activated NK cell lysis of tumor target cells. Similar to GA effect, such enhancement of cytotoxicity was abrogated by pretreatment with anti-CCR10. More work is needed to explore in details such an exciting possibility involving chemokines/chemokine receptors axis in mediating not only the migration of NK cells but also other activities such as cytotoxicity.

We consider the most important aspect of this work is the finding that drugs for MS such as GA and DMF as well as MMF upregulate the expression of CCR10 on the surface of IL-2-activated NK cells. Such finding might have potential relevance for treating cancer, particularly those tumors that secrete chemokines, which bind CCR10, including malignant melanomas and squamous cell carcinomas, which secrete CCL27 (47, 49), or colorectal cancer cells that secrete CCL28 (50). This is particularly relevant when one considers that there is an estimated 2–5 × 10^9^ NK cells in 5 L of blood. A 1% of this NK cell number represents a subset of NK cells that expresses CCR10 (an estimated number of 20–50 × 10^8^/5 L of blood). A 10% increase after treatment with the drugs described in this manuscript suggests that the number of CCR10^+^ NK cells might be increased to about 200–500 × 10^8^/5 L of blood; a substantial number of killer cells that may potentially inhibit tumor growth. Our next step is to examine in tumor-bearing animals whether these CCR10^+^-activated killer cells may migrate into the sites of colorectal cancers or melanomas after pretreatment with GA, MMF, or DMF. A number of cancers are, at present, incurable. For others, chemotherapy is only partially effective, and a significant proportion of patients relapse following treatment. Some hematological malignancies are treatable by hematopoietic stem cell transplantation (HSCT), but fewer than 30% of patients requiring HSCT have a suitable donor. NK cells possess the ability to spontaneously lyse certain target cells, including tumor cells. The major issue that faced investigators in this field is the inability of NK cells to migrate toward sites of tumor growth. This, despite the existence of more than 600 approved clinical trials using NK cells to treat various cancers. Our present work may provide a novel approach of harnessing activated NK cells *in vitro* for the purpose of administering into cancer patients. We propose that these cells can be harnessed *in vitro* with drugs such as GA, DMF (or MMF). These drugs perform two important functions for these cells; first, they increased their cytotoxicity against tumor cells, and second, they upregulate the expression of CCR10 on their surfaces. Consequently, NK cells can be targeted toward the growth of tumor cells that secrete CCL27 and CCL28.

## Conclusion

We previously described the effects of GA on chemokine receptor expression in an MS patient receiving this drug (51). However, this is the first demonstration that GA, DMF, and MMF upregulate the expression of an important chemokine receptor, i.e., CCR10, on the surface of activated NK cells. Such activity might have important implications in NK cell immunotherapy. Because immunotherapy using NK cells suffers from the inability of these cells to migrate into tumor stroma, it is now feasible to direct these cells toward sites of tumor growth, particularly those secreting CCL27 and CCL28. Hence, we suggest the feasibility of using such approach to treat cutaneous squamous cell carcinomas and squamous melanoma, as well as colorectal cancer.

## Author Contributions

AAM designed the experiments, performed the chemotaxis assays, analyzed the data, and wrote the paper. KS and ZA-J performed the flow cytometric experiments and other assays.

## Conflict of Interest Statement

The authors declare that the research was conducted in the absence of any commercial or financial relationships that could be construed as a potential conflict of interest.

## Supplementary Material

Figure S1 **Expression of CD56 molecule on the surface of IL-2-activated NK cells. (A)** Activated NK cells were labeled with either mouse FITC-conjugated isotype IgG or mouse FITC-conjugated anti-CD3 antibody. **(B)** Activated NK cells labeled with mouse PE-conjugated isotype IgG or mouse PE-conjugated anti-CD56 antibody. **(C)** Activated NK cells labeled with both FITC-conjugated anti-CD3 antibody and PE-conjugated anti-CD56 and examined in the flow cytometry. One of the four representative experiments was performed.

Figure S2 **IL-2-activated NK cells release CCL27 and CCL28 and express CD107a. (A)** NK cells were either left intact or were incubated for 24 h with 0.1, 1 or 10 μg/mL GA. Supernatants were collected, and the levels of CCL27 were measured. **(B)** CCL28 levels were evaluated using ELISA assay. **(C)** A representative experiment showing histograms for the expression of CD107a after incubating overnight with 10 μg/mL GA, 10 ng/mL CCL27, or 10 ng/mL CCL28 (left panel), or 10 μg/mL GA either alone or in combination with 10 ng/mL CCL27 or 10 ng/mL CCL28 (right panel). **(D)** Activated NK cells (1 × 10^6^/mL) were either untreated (–) or incubated overnight with 10 μg/mL GA (GA), 100 ng/mL CCL27, 100 ng/mL CCL28, or a combination of 10 μg/mL GA with 100 ng/mL CCL27 (GA + CCL27), or 100 ng/mL CCL28 (GA + CCL28). The cells were washed, and the expression of CD107a was evaluated by flow cytometric analysis. Mean ± SEM of values from three different donors. The binding of isotype control antibody is also shown.

Figure S3 **GA or supernatants collected from IL-2-activated NK cells increase the percentages of NK cells expressing Granzyme B**. IL-2-activated NK cells were incubated for 24 h with either media alone or with 10 μg/mL GA. In both treatments, the cells were incubated with supernatants collected from IL-2-activated NK cells. In other cultures, the cells were pretreated with the supernatants in the presence of 1 μg/mL mouse IgG isotype control for anti-CCL27 and anti-CCL28, or with 1 μg/mL of neutralizing mouse anti-CCL27 or mouse anti-CCL28. Upper panels show expression of Granzyme B in the absence of GA, whereas lower panels show expression of the same molecule in the presence of 10 μg/mL GA. One of the two representative experiments was performed. Percentages of positive cells are shown between brackets.
