# Supplementary figures and images for: Glatiramer Acetate, Dimethyl Fumarate, and Monomethyl Fumarate Upregulate the Expression of CCR10 on the Surface of Natural Killer Cells and Enhance Their Chemotaxis and Cytotoxicity
Source: Front Immunol. 2016 Oct 19;7:437. doi: 10.3389/fimmu.2016.00437 (PMC5069502; doi:10.3389/fimmu.2016.00437)

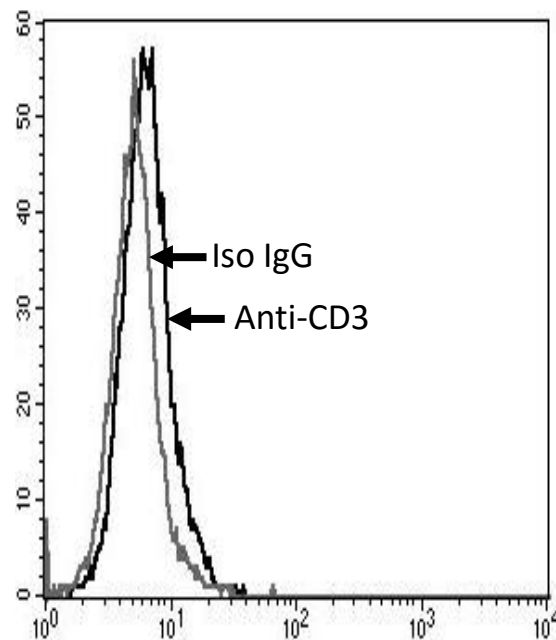

Mean Fluorescence Intensity

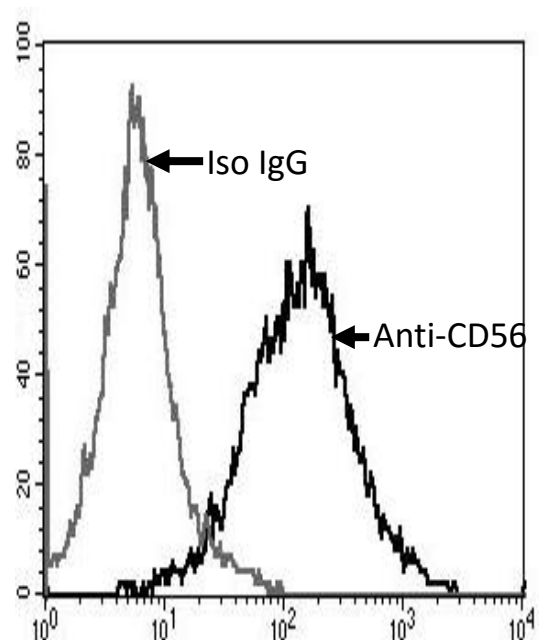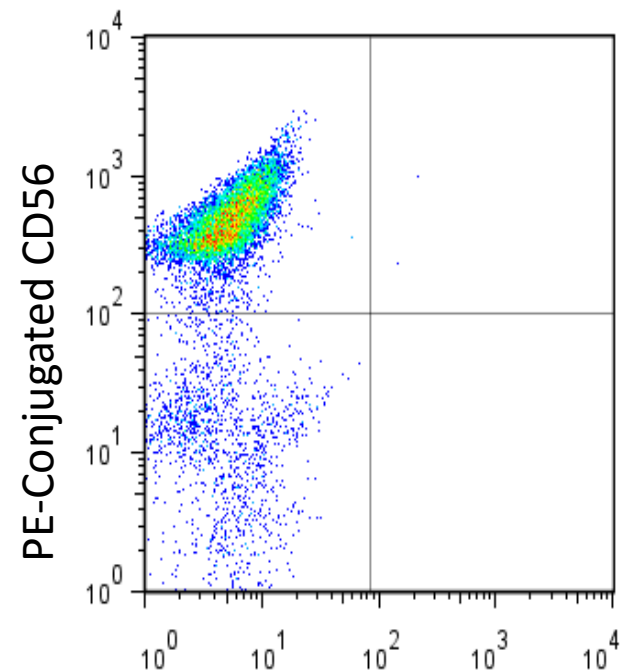

FITC-Conjugate CD3

Supplement: Figure S1 — Expression of CD56 molecule on the surface of IL-2-activated NK cells. (A) Activated NK cells were labeled with either mouse FITC-conjugated isotype IgG or mouse FITC-conjugated anti-CD3 antibody. (B) Activated NK cells labeled with mouse PE-conjugated isotype IgG or mouse PE-conjugated anti-CD56 antibody. (C) Activated NK cells labeled with both FITC-conjugated anti-CD3 antibody and PE-conjugated anti-CD56 and examined in the flow cytometry. One of the four representative experiments was performed. [file Image_1.PDF]

A

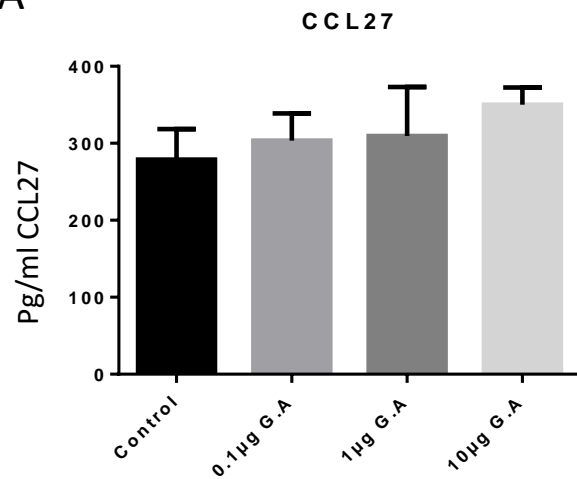

B

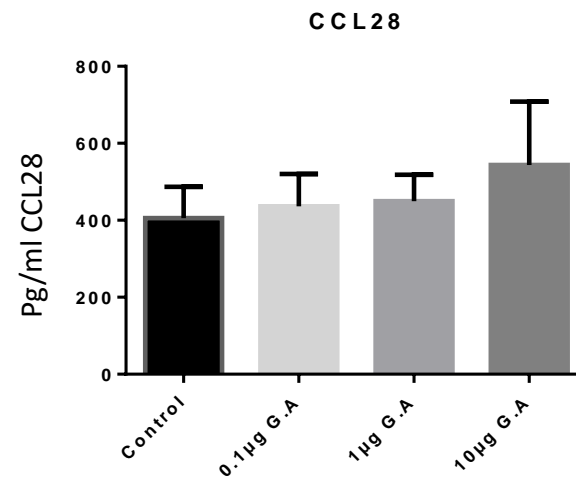

C

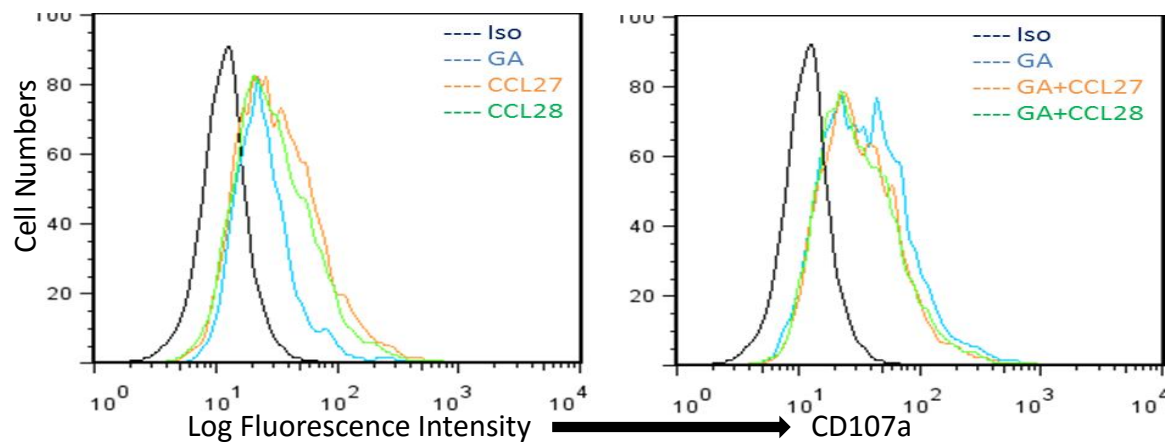

D

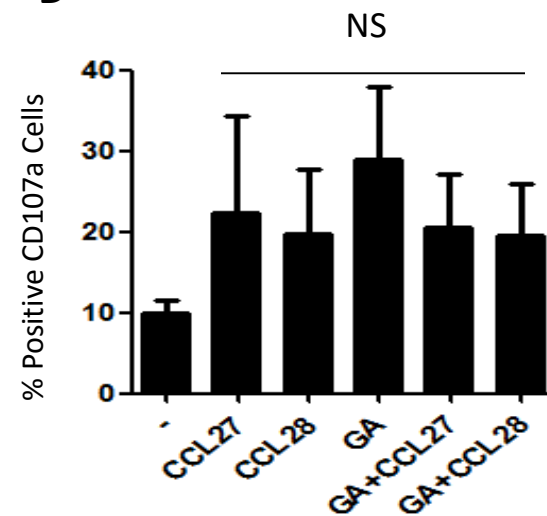

Supplement: Figure S2 — IL-2-activated NK cells release CCL27 and CCL28 and express CD107a. (A) NK cells were either left intact or were incubated for 24 h with 0.1, 1 or 10 μg/mL GA. Supernatants were collected, and the levels of CCL27 were measured. (B) CCL28 levels were evaluated using ELISA assay. (C) A representative experiment showing histograms for the expression of CD107a after incubating overnight with 10 μg/mL GA, 10 ng/mL CCL27, or 10 ng/mL CCL28 (left panel), or 10 μg/mL GA either alone or in combination with 10 ng/mL CCL27 or 10 ng/mL CCL28 (right panel). (D) Activated NK cells (1 × 10^6^/mL) were either untreated (–) or incubated overnight with 10 μg/mL GA (GA), 100 ng/mL CCL27, 100 ng/mL CCL28, or a combination of 10 μg/mL GA with 100 ng/mL CCL27 (GA + CCL27), or 100 ng/mL CCL28 (GA + CCL28). The cells were washed, and the expression of CD107a was evaluated by flow cytometric analysis. Mean ± SEM of values from three different donors. The binding of isotype control antibody is also shown. [file Image_2.PDF]
