# Supplementary material for: Glatiramer Acetate, Dimethyl Fumarate, and Monomethyl Fumarate Upregulate the Expression of CCR10 on the Surface of Natural Killer Cells and Enhance Their Chemotaxis and Cytotoxicity
Source: Front Immunol. 2016 Oct 19;7:437. doi: 10.3389/fimmu.2016.00437 (PMC5069502; doi:10.3389/fimmu.2016.00437)
Supplement: Figure S3 — GA or supernatants collected from IL-2-activated NK cells increase the percentages of NK cells expressing Granzyme B. IL-2-activated NK cells were incubated for 24 h with either media alone or with 10 μg/mL GA. In both treatments, the cells were incubated with supernatants collected from IL-2-activated NK cells. In other cultures, the cells were pretreated with the supernatants in the presence of 1 μg/mL mouse IgG isotype control for anti-CCL27 and anti-CCL28, or with 1 μg/mL of neutralizing mouse anti-CCL27 or mouse anti-CCL28. Upper panels show expression of Granzyme B in the absence of GA, whereas lower panels show expression of the same molecule in the presence of 10 μg/mL GA. One of the two representative experiments was performed. Percentages of positive cells are shown between brackets. [file Image_3.PDF]

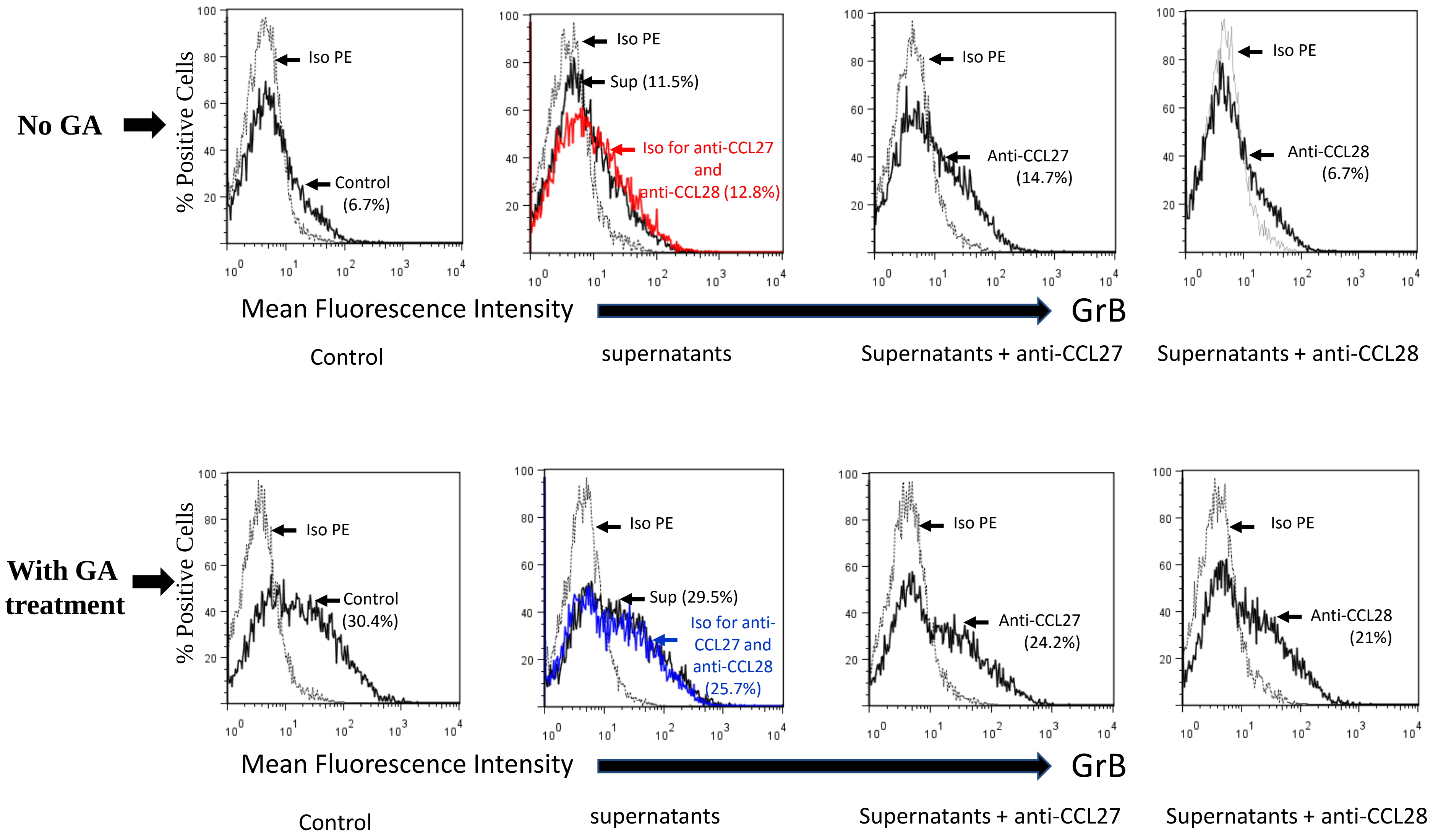
No GA
With GA treatment
% Positive Cells
Iso PE
Control (6.7%)
Sup (11.5%)
Iso for anti-CCL27 and anti-CCL28 (12.8%)
Anti-CCL27 (14.7%)
Anti-CCL28 (6.7%)
Control (30.4%)
Sup (29.5%)
Iso for anti-CCL27 and anti-CCL28 (25.7%)
Anti-CCL27 (24.2%)
Anti-CCL28 (21%)
Mean Fluorescence Intensity
GrB
Control
supernatants
Supernatants + anti-CCL27
Supernatants + anti-CCL28
